# Myanmar Articulation, Resonation, Nasal Emission, and Nasal Turbulence Test: A Preliminary Study

**DOI:** 10.1055/s-0043-1771522

**Published:** 2023-10-05

**Authors:** Kalyanee Makarabhirom, Benjamas Prathanee, Ampika Rattanapitak

**Affiliations:** 1 Department of Communication Sciences and Disorders, Faculty of Medicine, Ramathibodi Hospital, Mahidol University, Bangkok, Thailand; 2 Department of Otorhinolaryngology, Faculty of Medicine, Khon Kaen University, Khon Kaen, Thailand; 3 Eastern Languages Department, Faculty of Humanities, Chiang Mai University, Chiang Mai, Thailand

**Keywords:** speech disorders, Burmese, cleft palate

## Abstract

**Background** This article describes the development of the Myanmar Articulation, Resonation, Nasal Emission, and Nasal Turbulence test for children with cleft lip and palate (CLP), and evaluation of its validity and reliability.

**Methods** It was created by three Thai researchers and a Burmese research assistant based on Burmese phonology. The content validity was evaluated by six Burmese language experts. All test items were divided into three groups: high-pressure oral consonants, low-pressure oral consonants, and nasal consonants.

**Results** All items (58-word and 32-phrase/sentence) gave an excellent level of the expert agreement (item-level content validity indexes = 1.00). The target items were illustrated as color pictures. Each picture was clearly drawn and easy to identify. As a pilot study of face validity, all pictures were administered to 10 typical-developing children. The actual testing was assessed by 10 CLP children, and the developed test was analyzed through consultation of the Burmese teachers and interpreters from a speech camp. Testing scores for a total including three groups of target items were shown acceptable for internal consistency reliability (ranged from 0.4 to 0.88).

**Conclusion** The constructed test is valid in terms of its content.

## Introduction

The incidence of cleft lip and palate (CLP) is approximately 0.11 to 2.49 per 1,000 live births. 1 2 In Myanmar, congenital malformations in children with CLP are 0.6%, with congenital malformations estimated at approximately 60,000 cases per year. 3 Speech and resonance problems in children with CLP may remain even in cases when their lips and/or palates have been repaired. An estimated 88.56% (95% confidence interval [CI] = 84.47–92.65) of CLP children still have difficulties with articulation. Furthermore, approximately 43.26% (95% CI = 36.58–49.93) of them produce hypernasal speech. 4 These problems need to be addressed before children enter school so that age-appropriate speech can be produced and compensatory articulation errors do not develop. 5

Myanmar is a country with many ethnic groups in terms of culture, tradition, and language. There are more than 100 dialect languages, including among others Tai Yai, Karen, Wa, Kachin, Chin, Mon (Mon–Khmer), Tai Lue, Akha, and Lahu. Besides, Burmese language is the official language used mainly in schools. The majority of the population (∼37 million) uses this language. 6

The Burmese language is composed of 32 initial consonants which are divided into plosive/stop (voiceless/aspirated/voiced stop), nasal (voiceless/voiced nasal), fricative (voiceless/aspirated/voice fricative), lateral (voiceless/voiced lateral), and approximants (voiceless/voiced labials, voiceless/voiced dental; Table 1 ). There are 7 basic vowels or plain vowels (+ neutral vowel/ə/) and 4 diphthongs which generally appear with nasalized and stopped syllables, including 3 tones (neutral, creaky, and heavy). Syllables in Burmese are divided into closed and open syllables. Closed syllable contains nasal vowel or ends with glottal stop. Syllables with plain vowels and nasal vowel carry all three tones, whereas closed syllables with glottal stop carry only creaky tone. 7 8

**Table 1:** Initial consonants of Burmese language with IPA in [-]

Place of articulation Manner of articulation			Bilabial	Dental	Alveolar	Palatal	Velar	Glottal
Plosive	Voiceless	Unaspirated	p [ph]		t [t]	c [ʨ]	k [k]	ʔ [ʔ]
		Aspirated	ph [p ^h^ ]		th [t ^h^ ]	ch [ʨ ^h^ ]	kh [k ^h^ ]	
	Voiced		b [b]		d [d]	j [ʤ]	ɡ [ɡ]	
Fricative	Voiceless	Unaspirated		θ [θ]	s [s]	sh [ʃ]		h [h]
		Aspirated			hs [s ^h^ ]			
	Voiced	Voiceless			z [z]			
Nasal	Voiced		m [m]		n [n]	ɲ [ɲ]	ŋ [ŋ]	
	Voiceless		hm [m̥]		hn [n̥]	hɲ [ɲ°]	hŋ [ŋ°]	
Lateral	Voiced				l [l]			
	Voiceless				hl [l̥]			
Approximant	Voiced		w [w]			y [j]		
	Voiceless		hw [w̥]					

Abbreviation: IPA, International Phonetic Alphabet.

Perceptual assessment of articulation and resonance disorders is often cited as the gold standard. 5 9 However, assessment protocols using standardized tests would be better. To date, there are no articulation and resonance tests developed by speech-language pathologists (SLPs) in Myanmar because there are so few of these professionals in the country. Given the great impact of speech difficulties, particularly in CLP children, they should be assessed using standardized tests. Thus, there is a need to develop a standardized articulation, resonance, nasal emission, and turbulence test for assessment of speech outcomes in Burmese children with CLP.

The test should consist of simple pictures which cover all Burmese initial consonants and vowels at the word and sentence level.

In Myanmar, a Burmese articulation test has been developed to assess children's articulation. 10 This assessment has only word-level stimuli and does not focus on compensatory articulation difficulties including resonance disorders which typically appear in children with CLP. Articulation and resonance tests have been developed in some western countries, such as the Cleft Audit Protocol for Speech-Augmented 11 and the assessment tools of the Scandcleft speech groups. 12 Currently, Thailand is working toward developing a standardized test for articulation and resonance disorders at the level of words and sentences (Thai Universal Parameters of Speech Outcomes for People with Cleft Palate). 9 An advantage of this standardized test is that it can be used to evaluate the variation associated with speech and resonance disorders simultaneously. Our research team had previously created and used a similar test to evaluate articulation and resonant disorders in cleft palate children who enrolled for speech camps both in Thailand with the Thai version 13 and in Lao People's Democratic Republic with the Laotian version. 14 We found that these tests were able to contribute perceptual assessment in cleft children in identifying cleft speech type characteristics particularly well in compensatory articulation disorders. Consequently, as a research team, we set out to construct and develop an articulation, resonation, nasal emission, and nasal turbulence test in Burmese language to evaluate misarticulation and resonance disorders in children with CLP. The purpose of this study was to develop, validate, and determine internal consistency reliability of the test items of such a Myanmar Test.

## Methods

### Test Construction

After approval from the Ethical Committee of the author's hospital (ID 09–60–51), the researchers started to construct the articulation, resonation, nasal emission, and nasal turbulence test. The team consisted of (1) two Thai senior SLPs, (2) one Thai-Burmese linguist, (3) one Burmese research assistant, a native Burmese who fluently spoke and wrote Thai language, and (4) one English teacher, a native Burmese who had Master Degree in English language from Thai University. Both of the native Burmese were Northern Women's Development Foundation's staff.

Due to language and cultural differences across the country, it is not possible to apply the existing articulation tests among other languages for Burmese. Therefore, a picture-naming test of 58 items at the word level and 32 items at phrase or sentence level for the Myanmar Articulation, Resonation, Nasal Emission, and Nasal Turbulence test was created on the basis of Burmese sound system including concept and criteria of articulation and resonance test in previous study, 9 15 in that to use intraoral high pressure-sensitive phonemes such as plosive, fricative, and affricate consonants with controlling the impact of nasal vowels to determine an adequacy of velopharyngeal function. Our target sounds were divided into three groups: high-pressure oral consonants, low-pressure oral consonants, and nasal consonants.

In addition, the selected words of this study are representatives for 32 significant consonant phonemes with a wide range of places of articulation in Burmese phonemes ( Table 1 ). Its vowel system is composed of high vowel, low vowel, and nasal vowel. To avoid the influences from nasal vowel on consonant sound, words with basic vowels, fixed tones on each lexical item and closed syllable with glottal stop, were chosen. The selection of controlled stimuli (word and vocabulary) was also picked up based on the phonological noncomplexity and the most frequent, familiar, culturally based and visually transparent words. It was done on the criteria of children's vocabulary size and their phonological abilities. These were obtained from vocabularies of the Burmese language, textbooks at elementary level, 16 17 or original terms of the Burmese language including official words or language usage in school. The test consists of target phonemes in word level and phrase or short sentence levels that represented different Burmese consonants. This study focuses on the development of the standardized articulation, resonation, nasal emission, and nasal turbulence test in Burmese version. Regarding to its purpose whether it can be used to assess misarticulation and abnormal resonance in CLP children. Therefore, the psychometric properties of this test must be evaluated first.

### Validity Assessment

After a list of the target phonemes (58 items at the word level and 32 items at the phrase or sentence level) was developed, six Burmese linguistic experts reviewed and made comment for the content validity. A proper of the test which relevant to and representative of the constructed target sounds were designed to measure. The experts evaluated the consonants and vowels of those three groups of the target phonemes appearing in each item of the selected words and sentences.

All items were rated using binary scales (0 = not relevant, 1= good relevant). An item-level content validity index (I-CVI) was computed as the number of all experts giving a rating of 1 (all relevant scales) divided by the total number of expert participants.

Some target phonemes were adjusted according to expert advice. Then, all the selected vocabulary words were illustrated using clear, color pictures that were easy to identify and also allowed the children to make the target phonemes which the examiner need to test. Each picture corresponded to one target sound. Totally, there were 58 pictures at the word level and 32 pictures at the phrase or sentence level.

### Sample Testing

It is a try-out step with an individual volunteer to indicate each picture that is understood and compare with his/her experience that is relevant to that target phoneme we need to test, and discuss with his/her words or language that the volunteer can clearly understand. To identify in terms of its content that lead to revise for improvement.

A research team trialed the test and evaluated its face validity. The assessment was administered to 10 typical-developing children (6 boys and 4 girls) aged 7.0 to 13.0 years old, attending a private school in Tachileik, Myanmar. The children were passed from screening for speech and language development. They were asked to name pictures at the word and sentence levels. An elementary school teacher and a research assistant acted as interpreters to assist on data collection. Both interpreters were graduate volunteers from Myanmar, fluent in Burmese as well as Thai. If the children could not correctly name a picture or did not know what the picture was, they were asked again to list characteristics of the pictures or describe features (e.g., size, shape) that would help elicit each target word and sentence. After the face validity testing, some pictures were revised and redrawn following the children's, speech assistants', and research team's suggestion. A constructed process was discussed and made consensus among the research team until validity was reached at 0.8. All of the children could easily imitate or read the items in the pilot study.

### The Actual Testing

The researcher selected the picture samples representing the target phonemes to be measured and revised them to be clear and easy to understand, but still maintaining the characteristic of the test measurement in terms of their high-pressure oral consonants, low-pressure oral consonants, and nasal consonants.

As a construction of the test, the words representing all consonant and vowel phonemes in Burmese language including colloquial short phrase or sentences were implemented by the research team. The actual testing was assessed by 10 CLP children (6 boys and 4 girls) aged 6.0 to 14.0 years old who participated in the speech camp at Mahamuni Monastery, Tachileik Province, Eastern Shan State, Myanmar ( Table 2 ). The developed test was analyzed through consultation of the Burmese teachers and interpreters from a speech camp. Again, those unclear pictures were jotted down and reviewed. Several percentage of phonemes (consonants and vowels) errors were identified as seen in our separate study about speech outcomes. 18

**Table 2:** Demographic characteristics and perceptual assessment in children with CLP

No.	Gender	Age (y)	Diagnosis	Palatal repair method	Oronasal fistula (ONF) size/site	Hyper nasality	Voice problem	Language usage at home/school
C1	Male	11	Bilat. CLP	2 Flap palatoplasty	−	Moderate	Hoarse/breathy	Shan/Burmese
C2	Male	7	CP	2 Flap palatoplasty	Medium (3–5 mm)/anterior hard palate	Mild	Hoarse	Shan/Burmese
C3	Female	14	CP	Double opposing Z-palatoplasty	−	Moderate	Hoarse/breathy	Lahu/Burmese
C4	Female	7	Lt. CLP	2 Flap palatoplasty	Large (> 5 mm)/lingual alveolar	WNL	Normal	Burmese
C5	Male	6	Rt. CLP	2 Flap palatoplasty	−	WNL	Hoarse	Lahu/Burmese
C6	Male	11	Bilat. CLP	2 Flap palatoplasty	−	WNL	N/A	Shan/Burmese
C7	Male	12	CP	N/A	−	Mild	Hoarse	Burmese
C8	Male	8	Rt. CLP	N/A	−	Mild	Hoarse	Mandarin/Burmese
C9	Female	6	Lt. CLP	Double mucosal flaps	−	Mild	Hoarse	Burmese
C10	Female	10	Rt. CLP	2 Flap palatoplasty	Small (< 2 mm)/hard palate	Moderate	Hoarse/breathy	Burmese

Abbreviations: CLP, cleft lip and palate; CP, cleft palate; Bilat., bilateral; Lt., left; N/A, not available; Rt., right; WNL, within normal limit.

According to the number of naming responses from the three groups of picture-naming test which represent different target sounds (high-pressure oral consonants, low-pressure oral consonants, and nasal consonants) were dichotomous responses (correct and incorrect), then a correlation among the items of the test in the word level was obtained for internal consistency reliability to study the measurement properties of the test by means of Kuder–Richardson (KR-20). ^19^ Purposive samples testing for reliability were done in 10 CLP children. A protocol in this stage was the same as in a try-out step.

## Results

The Myanmar Articulation, Resonation, Nasal Emission, and Nasal Turbulence test consists of two levels: word and phrase or sentence levels. At the word level, there were 58 items with initial target sounds covering all Burmese consonants combined with low and high vowels which were not nasal vowels. At the phrase or sentence level, there were 32 items using initial consonants parallel with the word level. A list of these words and sentences were arranged from low-pressure oral sounds (/w/, /y/, /l/) to high pressure-sensitive oral target sounds as well as nasal sounds to assess misarticulated phonemes and the presence of hyper- or hyponasality in a subject's speech ( Table 3 ).

**Table 3:** List of the initial consonant sounds of the Myanmar test at the word and sentence level

We assessed content validity of the Myanmar Articulation, Resonation, Nasal Emission, and Nasal Turbulence test using qualitative evidence. The consonants and/or vowels of each item and the selected vocabularies from a constructed list were evaluated by six Burmese linguistics experts. Fifty-five of 58 items (word level) and 25 of 32 items (phrase/sentence level) of the constructed test had statistically nonsignificant differences, as found by expert opinions (I-CVI of 1.00), indicating an excellent level of agreement. Only few of the target lists of our constructed test were adapted as recommended by the experts. All picture-naming tests proved the content validity of this developed test.

Next in a try-out step, our test was applied with 10 typical children from a selected school. The result revealed that more than 80% of the children named all pictures correctly.

Some confused pictures, for example, /hwɛ́ʔ/(hide), /du/ (knee), /hôuʔ/ (yes), /kha/ (waist), and /mo/ (rain), were redrawn according to the Burmese teachers, interpreters from a speech camp, and the children's suggestions. In each picture, the research team consensus was reached in accordance with its target sound and meaning. Finally, a completed version of the Myanmar Articulation, Resonation, Nasal Emission, and Nasal Turbulence test was achieved. The final version of the test includes 90 items (consonant and vowel singleton words and sentences).

At the stage of actual testing, the test was administered to 10 CLP children who were attending a speech camp. Their screening of speech and language characteristics is summarized in Table 2 .

The result of our developed articulation test demonstrated that the most common type of articulation errors were functional articulation disorders (90%) in a pattern of substitution (voiceless for voice sounds [50%], nasalized voice pressure consonant [30%], nasal consonant for oral pressure consonant [20%], and glottalized sounds [20%]). 18 Those have also shown that the children had different numbers and characteristics of speech disorders according to the degree of severity of their problems, such as C4 and C2 had the most severe and severe speech problems, respectively. Regarding the palatal surgery, patients C7 and C8 had received from another hospital, not the same place as a group of the samples. Therefore, the researcher could not show information about the palatal repair method. Although this group of children received the same palatal repair method, the degree of severity of the problem was different, thus affecting different speech outcome. 18

Statistical analysis was further applied to identify the internal consistency reliability of the test. It was studied with a target sample of 58 phonemes (word level) divided into three groups: high-pressure oral consonants, low-pressure oral consonants, and nasal consonants. A reliability coefficient of 0.7 was obtained for the total of 58 phonemes that make up the test's stimuli. Where 0.71, 0.40, and 0.88 were the reliability coefficients for high-pressure oral consonants, low-pressure oral consonants, and nasal consonants, respectively ( Table 4 ). A low coefficient reliability (0.4) indicated a weak relationship between items on the test. It may be that pictures representing sounds in low-pressure oral consonants filed to convey to the children because the Burmese language has many nasalized sounds. Thus, there were few or limited numbers of those appropriated picture items. Although a group of nasal consonants and a total of the target sound picture items presented a high (0.88) and quite high (0.7) internal consistency reliability among the items of the constructed test, demonstrating an acceptable value. By means of Kuder–Richardson reliability coefficient, it needs to be more than 0.75 to be acceptable. 19 Consequently, our developed test met a minimal reliable criterion to be a good tool.

**Table 4:** An internal consistency reliability of the Myanmar Articulation, Resonation, Nasal Emission, and Nasal Turbulence test in 10 CLP children, using the Kuder–Richardson reliability test (KR-20)

Group of pictures naming test, representing different target sounds	Picture of different target sounds sample size	Number of CLP children	Number of correct naming responses	Number of incorrect naming responses	Variance ( *S* ^2^ )	KR-20 reliability coefficients
High-pressure oral consonants	34	10	305	35	2.61	0.71
Low-pressure oral consonants	13	10	111	19	1.38	0.4
Nasal consonants	11	10	92	18	5.5	0.88
Total of target sounds	58	10	508	72	2.97	0.7

Abbreviation: CLP, cleft lip and palate.

**Fig. 1:**
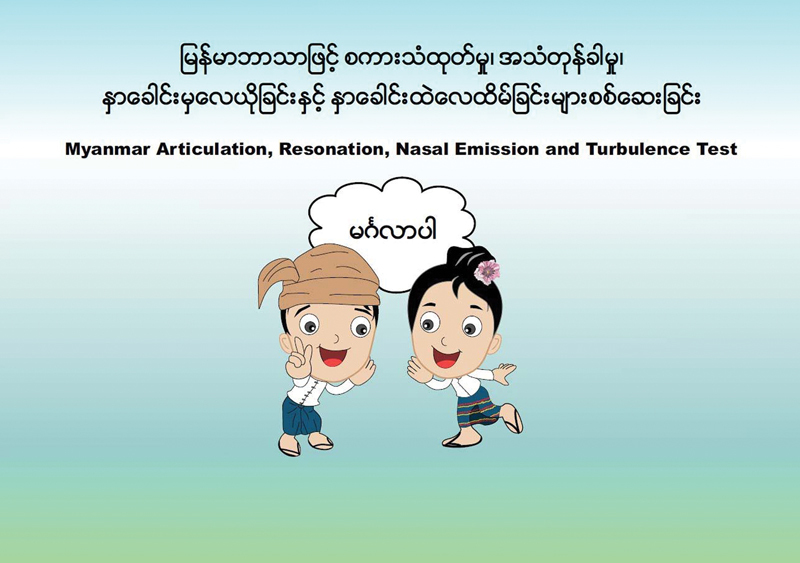
The Myanmar Articulation, Resonation, Nasal Emission, and Nasal Turbulence test.

**Fig. 2:**
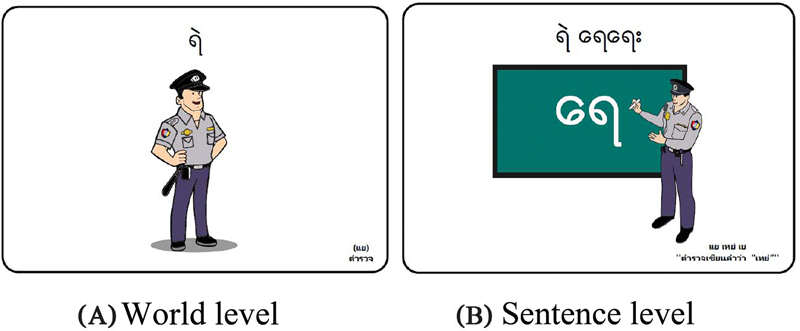
Examples of pictures presenting target sounds at the word and sentence level.

Eventually, the researchers achieved a completed version of the Myanmar Articulation and Resonation test judged as sufficiently valid in terms of its content and proved its internal consistency reliability ( Figs. 1 and 2 ).

## Discussion

The researchers developed the Myanmar Articulation, Resonation, Nasal Emission, and Nasal Turbulence test to assess misarticulated sounds and the presence of resonance disorders in children's speech. In terms of word structure, a variety of consonant place and manner sequences were used as well as taking vocabulary and different contexts into account. The words covered a range of areas, were familiar to children, and could be represented by color pictures. 20 Short sentences or phrases were shaped into structures to reflect their high pressure-sensitive oral consonants. A few words were then selected from the elicited lists to create test items including the name of a television cartoon show (“bò-bò”); a person's name (e.g., “shè-shè,” “zɔ̀-zɔ̀,” “pâuʔ-pâuʔ,” and “hnun-hnun”); and exclamations like laughter (“há há há”). Some required a leading phrase or sentence to elicit a response with the desired target sounds, that is, “hŋɛ́ʔhŋa” (bird rental). Some structures could not be formed into a phrase or sentence, in which case the researchers then presented them in the form N + N + N, that is, “hsì hse hsa” (oil, drug, salt). In some structures we had to delete an adjacent consonant in phrases, that is, “ɡù də-ɡa” (cavern gate).

In this case, the SLP would need to pay attention only to the target sounds. In addition, high-frequency usage of the same sound or word at the phrase or sentence level, as well as the word level, helped the children recognize and identify the picture faster. This increased the efficiency with this tool in the testing process. The content of the constructed test is shown in Table 3 .

The consonants were divided into three groups and then sorted as follows: low-pressure oral sounds (i.e., glide, lateral), high-pressure oral consonants (i.e., plosive, affricate, fricative), and nasal consonants. Each consonant group was combined with either a low or a high vowel except nasal vowels. The high-pressure oral consonants were used to assess the presence of hypernasality whereas nasal consonants were used to assess the presence of hyponasality. 5 9 21 The high-pressure oral consonants, particularly voiceless sounds, had the greatest height of velar contact and maintained velopharyngeal closure. 21 Phonological aspects of the Burmese language are different from other languages.

Features of the language include degrading of final consonants, aspiration, and partial devoicing as indicated with an initial /h/ preceding the corresponding consonants, for example, /ny/, /hny/, and /ng/, which are not consonant clusters but transcribed as clusters for convenience in Burmese tradition. 7 This language has a larger number of, or more frequently occurring, high-pressure consonants (i.e., fricatives, stops, and affricates) and more voiced stop consonants ( Table 1 ).

Both high-pressure consonants and voiced stop consonants which perceiving as more hypernasal sounds were more affected by velopharyngeal dysfunction especially in people with cleft palate than those with low-pressure consonants. Aside from vowel height, the presence of glottal stop /ʔ/ in the phonemic inventory of a language plays a role in the presence and perception of nasality in speech where it is more increased. 5

In addition, the Burmese language has many nasalized sounds. It also has a pattern of voiceless nasal and glottal fricative [h̃], a sound that is described as associated with nasalization, and is uncommon in the world's languages. Voiceless nasal consonants are mostly found in the languages of Southeast Asia, particularly in ethnic people. 22 At least eight major national ethnic groups were found in Myanmar. Further interesting features of the Burmese language are the open and glottalized syllables which have placements out of the oral cavity. 7

As noted earlier, compensatory articulation errors were found in children with CLP. They used more words with nasals, glides, and glottals than words with oral consonants. Glottalization and nasalization are the predominant error patterns, describing the precise phonetic characteristics of a type of cleft palate speech. 4 5 9 11 A focus on these features means SLPs may struggle to reach a clear diagnosis of speech difficulties and nasalization.

In terms of content validity, the newly constructed test was reviewed by a panel of six Thai-Burmese linguistics experts based on requirements of relevance, simplicity and clarity. I-CVIs of 1.00 were shown for measurement of all individual items, including all consonants of the target syllables in the initial positions along with all vowels except nasal vowels. This indicates an excellent level of expert agreement. In the current study, there were five unclear pictures requiring correction in a trial content validity administration.

According to Polit and Yang, 19 a coefficient between 0.4 and 0.88 like the one we found, is considered to indicate low to acceptable internal consistency reliability. This result reflected at least approval for our constructed test items. Although we strongly support the test to be a good tool, a further study with a large sample size should be conducted to proof the consistency with test–retest reliability.

Ultimately, the researchers obtained a complete articulation and resonation test, which found to be sufficiently valid in terms of its content and properly acceptable internal consistency in terms of its reliability. The target words are appropriate for the test objectives, specifically for the analysis of Burmese phonology. It can be used to evaluate the speech of children with CLP as well as could also be easily adapted to use for other Burmese people with another type of articulation disorders.

Moreover, this study focuses on the test construction in the Burmese language version, which is the invention of words that were picture-naming tests performed to evaluate the articulation, resonation, nasal emission, and nasal turbulence in Myanmar. Thus, we had to select all appropriate initial consonant sounds in this language system. Finally, we achieved initial target phonemes covering all Burmese consonants combined with low and high vowels which were able to assess the problems of articulation including resonation, nasal emission, and nasal turbulence as reported in Prathanee et al. 18 Particular phonemes with oral high-pressure sounds could detect the problems of hypernasality and/or nasal air emission due to velopharyngeal insufficiency. 11
